# Assessment of topical corticosteroid ointment on postcesarean scars prevention: A prospective clinical trial

**DOI:** 10.12669/pjms.35.2.553

**Published:** 2019

**Authors:** Elif Meseci

**Affiliations:** 1 *Elif Meseci, MD. Acıbadem Kozyatagı Hospital, Department of Obstetrics and Gynecology, Inonu Caddesi, Okur Sokak, No:20 Kozyatagi, 34742, Istanbul, Turkey*

**Keywords:** Cicatrix, Methyl prednisolonate, Topical administration, Wound healing

## Abstract

**Objective:**

To evaluate the effectiveness of corticosteroid ointment in hypertrophic scars prevention following Cesarean section.

**Methods:**

This study was conducted between June 2017-May 2018 in Acıbadem Kozyatagı Hospital. Sixty-one patients (31 treatment and 30 control patients) took part in the current study which evaluated wound outcomes and patient satisfaction. All patients’ wound characteristics were assessed via the modified Vancouver Scar Scale (MVSS) score (height, pigmentation, vascularity, and pliability) at baseline (post-op 10^th^ day), three months and six months. The treatment group received corticosteroid cream every other day for three months. Comparative evaluations and time-bound changes were evaluated in both groups.

**Results:**

The mean age of the subjects was 31.28 ± 3.95 years. While the height and vascularity subsection scores of corticosteroid recipients were significantly reduced compared to those without treatment at three months, the scores were similar at six months. Furthermore, pliability and pigmentation decreased equally in both groups. There was high satisfaction with scar healing in the experimental group (20%, n=6), while 12.9% (n=4) of the patients were satisfied in the control group. Two patients reported itching after treatment.

**Conclusions:**

The clinical outcomes in both groups were similar. Although vascularity and height parameters improved in three months, similar results were also observed in the group that did not receive treatment after the end of six months. This may have been due to the fact that treatment was stopped after three months. We recommend that the hypothesis be tested in larger series in future studies.

## INTRODUCTION

Post-operative surgical scars such as keloids and hypertrophic scars (HS) generally occur among persons with abnormal wound healing.^1^ Hypertrophic scars are usually characterized by the presence of inflammation, excess fibroblast proliferation, and abnormal deposition of extracellular matrix proteins.^2^

Cesarean section (CS) is one of the most common major surgical interventions carried out on the female population and postoperative scar development is not rare after the procedure. Scars do not only create problems from an esthetic point of view, they also increase the risk for infection and may also cause adverse symptoms such as itching, pain etc. These problems in post-natal women may lead to serious psychological problems in a patient group which is known to be predisposed to distress and depression.^3^,^4^ Therefore, even minor improvements in scar outcomes may relieve stress among new mothers.

Several treatment and prophylactic modalities including surgical excision, radiation, pressure therapy, cryotherapy, topical silicone gel, intralesional corticosteroid injections, interferons, fluorouracil, laser treatment, and many other medications have been used in the management of HS and keloids.^5^-^13^

The aim of our study was to determine the prophylactic efficacy of topical corticosteroid ointment as a non-invasive form of therapy for post-operative HS and keloids following Cesarean section.

## METHODS

The present prospective study was conducted between June 2017-May 2018 in Acıbadem Kozyatagı Hospital, Istanbul, Turkey. This study was in line with the Declaration of Helsinki and was approved by the hospital Ethics Committee. All patients were informed about the study and written consent was obtained.

Sixty-two patients who underwent a cesarean section through a primary Pfannenstiel incision were included. The patient cohort included were aged between 21–42 years and were determined to have type II-IV Fitzpatrick skin types. All patients’ wound closures were performed via the same procedure performed by the same surgeon. Subcutaneous tissues were closed with interrupted sutures (2/0 Caprosyn™ [polygytone 6211]). Skin was closed in continuous manner with 4/0 Monocryl™ (poliglecaprone 25). The exclusion criteria for the study were as follows: having a history of HS or keloids, having previously undergone surgeries which involved abdominal incisions, having a systemic infection, suffering from wounds with discharge or without visible signs of normal epithelialization, having chronic medical illnesses known to affect wound healing (such as diabetes mellitus, chronic renal failure, hematological disease, obesity), and finally, those with a known hypersensitivity/allergy to corticosteroids were excluded.

After confirming normal epithelialization and wound healing at the incision site on the 10^th^ postoperative day, patients who met the selection criteria were randomized to the experiment group or control group. Randomization was performed with SPSS 21 program. Patients were numbered and recorded in the program. Random number assignment was made for each patient using the RV Bernoulli (0.5) method. Zero codes were assigned to the control group and 1 codes to the experimental group. At the end of the analyses, 31 patients were included in the control group and 30 patients were included in the experimental group. In the experiment group (n=30), methylprednisolone cream (Advantan®; Intendis GmbH, Germany) was applied as a thin layer to the incision site twice a day, starting from postoperative day 10. However, in order to prevent skin atrophy, the applications were performed every other day. The treatment duration was three months. The control group received no treatment. A blinded dermatologist performed the follow-up examinations before treatment (at baseline, i.e. on the 10^th^ postoperative day), after three months of treatment, and three months after discontinuation of the treatment (at the end of the 6^th^ month). During the follow-up period, one patient from the experiment group was excluded because of wound dehiscence which occurred at the end of post-op 2^nd^ month. Therefore, a total of 61 patients (30 patients in the experiment group and 31 patients in the control group) were analyzed at the end of the 6-month follow-up period.

Scar assessment was performed using the modified Vancouver Scar Scale (MVSS) by assessing scar *pigmentation* (0: normal color, 1: hypo-pigmentation, 2: hyper-pigmentation), *vascularity* (0: normal color, 1: pink, 2: pink to red, 3: red, 4: red to purple, 5: purple), *pliability* (0: normal, 1: supple, 2: yielding, 3: firm, 4: banding-rope tissue, 5: contracture), and *height* (0: normal/flat, 1: <2 mm, 2: 2–5 mm, 3: >5 mm).^14^ A linear probe ultrasound (*General Electric Voluson730 Pro* Ultrasound, 5-17MHz, USA) was used to precisely measure scar thickness.

Side effects experienced by the patients were also assessed every two months. At the end of the 6-month follow-up period, patients in both groups were asked to rate their satisfaction using a 4-point grading scale (1=unsatisfied, 2=slightly satisfied, 3=satisfied, 4=very satisfied).

### Statistical Analysis

All analyses were performed with the SPSS v21 program. The normal distribution of numerical variables was checked with the Shapiro-Wilk test. Variables that conformed to normal distribution were calculated and reported as mean ± standard deviation (SD), while the remaining variables were calculated and reported as median (minimum – maximum). Categorical variables were given in frequency of occurrence and percentage. The Student t-test was used for cross-group comparisons of non-repeating measurements of continuous numerical data. Friedman’s two-way variance analysis was used to assess repeating measurements for the MVSS variable and this was compared with the Mann Whitney U test by obtaining the amount of change over time. In the evaluation of the categorical data measured repeatedly, Generalized Estimating Equations/GEE) and Chi-squared analyses were utilized. The cases where P values were 0.05 and below were considered statistically significant.

## RESULTS

Sixty-two patients were included in the study. One patient in the experiment group was excluded due to wound dehiscence and subsequent suspicion of infection at two months. A final group of 61 patients (30 patients in the experiment group and 31 patients in the control group) completed the planned duration of six months in the study. The mean age of subjects was 31.28 ± 3.95 years. There was no significant difference between the control and experiment groups (p=0.816).

As regard to height score, comparison of baseline (post-op 10^th^ day), 3^rd^ month and 6^th^ month scores revealed a significant decreased at the 3^rd^ month and 6^th^ month evaluations as compared with the baseline evaluation. There was no significant difference between the 3^rd^ and 6^th^ month evaluations. When the experiment and control groups were compared, height scores were significantly lower in the experiment group at the 3^rd^ month assessment (p=0.001), while there was no significant difference between the groups at the 6^th^ month assessment (p=0.163).

The pliability score was found to be decreased at the 6^th^ month evaluation as compared with baseline and 3^rd^ month evaluations in both groups. However, no significant differences were observed between the baseline and 3^rd^ month scores. When groups were compared, no differences were found in terms of scar pliability (Table-I).

**Table-I:** Descriptive statistics and p-values of the study.

	Total	Experiment group (n=30)	Control group (n=31)	p-values
Age	31.28 ± 3.95	31.4 ± 4.23	31.16 ± 3.72	0.816
BMI	25.65 ± 2.58	24.7 ± 2.67	26.57 ± 2.16	0.004
***Lesional Height***				
Baseline				
0	3 (4.9%)	2 (6.7%)	1 (3.2%)	0.080
1	33 (54.1%)	20 (66,7%)	13 (41.9%)	
2	25 (41.0%)	8 (26.7%)	17 (54.8%)	
3	0 (0.0%)	0 (0.0%)	0 (0.0%)	
3^rd^ month				
0	21 (34.4%)	17 (56.7%)	4 (12.9%)	0.001
1	27 (44.3%)	7 (23.3%)	20 (64.5%)	
2	13 (21.3%)	6 (20.0%)	7 (22.6%)	
3	0 (0.0%)	0 (0.0%)	0 (0.0%)	
6^th^ month				
0	34 (55.7%)	21 (%70.0%)	13 (41.9%)	0.163
1	13 (21.3%)	4 (%13.3%)	9 (29.0%)	
2	12 (19.7%)	4 (%13.3%)	8 (25.8%)	
3	2 (3.3%)	1 (3.3%)	1 (3.2%)	
p (intragroup)		0.001	0.002	
***Pliability***				
Baseline				
0	1 (1.6%)	1 (3.3%)	0 (0.0%)	0.255
1	18 (%29.5%)	11 (36.7%)	7 (22.6%)	
2	42 (68.9%)	18 (6.7%)	24 (77.4%)	
3	0 (%0.0%)	0 (0.0%)	0 (0.0%)	
4	0 (%0.0%)	0 (0,0%)	0 (0,0%)	
3^rd^ month				
0	6 (9.8%)	5 (16.7%)	1 (3.2%)	0.119
1	26 (42.6%)	14 (46.7%)	12 (38.7%)	
2	22 (36.1%)	7 (23.3%)	15 (48.4%)	
3	7 (11.5%)	4 (13.3%)	3 (9.7%)	
4	0 (0.0%)	0 (0.0%)	0 (0.0%)	
6^th^ month				
0	16 (26.2%)	11 (36.7%)	5 (16.1%)	0.149
1	24 (39.3%)	10 (33.3%)	14 (45.2%)	
2	11 (18.0%)	6 (20.0%)	5 (16.1%)	
3	9 (14.8%)	2 (6.7%)	7 (22.6%)	
4	1 (1.6%)	1 (3.3%)	0 (0.0%)	
p (intragroup)		0.019	0.032	
***Vascularity***				
Baseline				
0	0 (0.0%)	0 (0.0%)	0 (0.0%)	0.356
1	1 (1.6%)	1 (3.35)	0 (0.0%)	
2	17 (27.9%)	7 (23.3%)	10 (32.3%)	
3	36 (59.0%)	20 (66.7%)	16 (51.6%)	
4	7 (11.5%)	2 (6.7%)	5 (16.1%)	
3^rd^ month				
0	14 (23.0%)	12 (40.0%)	2 (6.5%)	0.015
1	23 (37.7%)	7 (23.3%)	16 (51,6%)	
2	17 (27.9%)	9 (30.0%)	8 (25,8%)	
3	6 (9.8%)	2 (6.7%)	4 (12.9%)	
4	1 1.6%)	0 (0.0%)	1 (3.2%)	
6^th^ month				
0	26 (42.6%)	15 (50.0%)	11 (35.5%)	0.097
1	23 (37.7%)	8 (26.7%)	15 (48.4%)	
2	10 (16.4%)	7 (23.3%)	3 (9.7%)	
3	2 (3.3%)	0 (0.0%)	2 (6.5%)	
4	0 (0.0%)	0 (0.0%)	0 (0.0%)	
p (intragroup)		<0.001	<0.001	
***Pigmentation***				
Baseline				
0	0 (0.0%)	0 (0.0%)	0 (0.0%)	0.255
1	3 (4.9%)	3 (10.0%)	0 (0.0%)	
2	58 (95.1%)	27 (90.0%)	31 (100.0%)	
3	0 (0.0%)	0 (0.0%)	0 (0.0%)	
3^rd^ month				
0	7 (11.5%)	4 (13.3%)	3 (9.7%)	0.092
1	16 (26.2%)	10 (33.3%)	6 (19.4%)	
2	35 (57.4%)	13 (43.3%)	22 (71.0%)	
3	3 (4.9%)	3 (1.0%)	0 (0.0%)	
6^th^ month				
0	7 (11.5%)	4 (1.33%)	3 (9.7%)	0.882
1	32 (52.5%)	15 (50.0%)	17 (54.8%)	
2	22 (36.1%)	11 (36.7%)	11 (35.5%)	
3	0 (0.0%)	0 (00%)	0 (0.0%)	
p (intragroup)		<0.001	0.015	
***MVSS***^(1)^				
Baseline	8 (4 - 10)	8 (4 - 9)^a^	9 (6 - 10)^a^	0.411
3^rd^ month	6 (0 - 10)	4 (0 - 10)^b^	6 (1 - 9)^b^	
6^th^ month	4 (0 - 11)	2,5 (0 - 11)^b^	4 (1 - 10)^b^	
p (intragroup)		<0.001	<0.001	
Satisfaction				
Unsatisfied	3 (4.9%)	2 (6.7%)	1 (3.2%)	0.663
Slightly satisfied	17 (27.9%)	9 (30.0%)	8 (25.8%)	
Satisfied	31 (50.8%)	13 (43.3%)	18 (58.1%)	
Very satisfied	10 (16.4%)	6 (20.0%)	4 (12.9%)	

BMI: body mass index; MVSS: modified Vancouver Scar Scale. (1) Same letters show that there are no significant differences among repetitive measurements.

In both groups, scar vascularity showed a trend of significant reduction over time. Vascularity decline in the experiment group was significantly more than the control group at 3^rd^ month evaluation (p=0.015). However, there were no significant difference between the two groups at 6^th^ month evaluation (p=0.097). Evaluation of pigmentation scores showed that 6^th^ month scores were significantly lower than baseline and 3^rd^ month scores in both groups; however, no significant differences were observed when groups were compared with each other.

**Fig.1:**
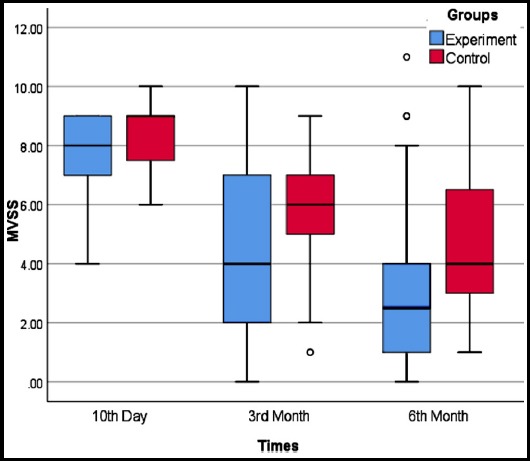
The distribution of MVVS scores of experiment and control group.

Comparisons of the 3^rd^ month and 6^th^ month total MVSS scores revealed that all scoring parameters (height, pigmentation, vascularity, pliability, and total MVSS score) significantly decreased across the board as compared to baseline evaluation in both groups. There was no significant difference between the 3^rd^ and 6^th^ month scoring values (Fig.1). There was no significant difference between experiment and control groups in terms of the amount of the decline in scores (p=0.411).

**Fig.2:**
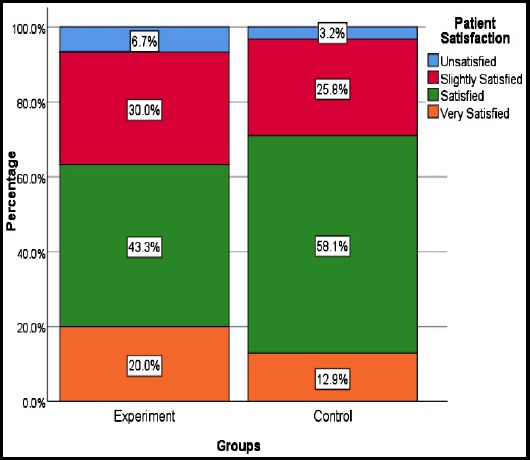
The distribution of satisfaction rates of experiment and control group.

Evaluation of patient satisfaction at the 6^th^ month follow-up demonstrated that the rate of ‘satisfied’ scores was significantly high in both groups, with no significant difference between the groups (p=0.663). After three months of using the methylprednisolone cream, 20% (n=6) of the experimental group and 12.9% (n=4) of the control group reported “high satisfaction” for scar-healing. In terms of adverse effects, itching was reported by two patients following the application of the cream (Fig.2). No other problems were reported.

## DISCUSSION

Wound healing is comprised of a few key steps; the inflammatory stage, tissue-formation, and remodeling.^15^ The precise pathophysiological mechanisms leading to scar formation remains elusive; however, available data suggests that fibroblast activity, extracellular matrix components, growth factors and cytokines possibly contribute to scar formation in addition to the roles of other mechanisms.^16^

We evaluated a topical corticosteroid cream versus no treatment to assess the effects on surgical scar formation, in terms of both efficacy and convenience. Topical administration of the corticosteroid in a cream formulation was preferred to mitigate the potential side effects seen with the conventional approach.^7^,^17^ Moreover, we predicted higher patient compliance with non-invasive, painless, and easily applied treatment.

Corticosteroids exert anti-inflammatory effects on the immune system and also act to decrease collagen and glycosaminoglycan synthesis whilst increasing collagen and fibroblast breakdown.^18^ Intralesional corticosteroid injection has been widely accepted and implemented in the management of HS and keloids.^7^-^10^,^17^,^19^ Observable reduction in the scar volume and an improvement in the scar pliability, height, and symptoms can be achieved with intralesional injection therapy. However, owing to poor tissue absorption, intralesional injections are usually preferred for mature scars.

Studies have shown that the topical use of corticosteroid cream is promising in HS management, with higher rates of satisfaction when compared to silicone-based controls.^20^,^21^ Initiation of intralesional corticosteroid injections are recommended to be reserved until the end of the 6^th^ month for patients with postoperative linear scars which could not be managed with prophylactic treatments (silicone gel, pressure treatment, moisturizing, taping), or in patients who have recurrent HS.^9^,^10^,^22^-^24^

We aimed to evaluate a novel approach where corticosteroid cream was used in the early post-operative period during which tissue absorption is favorable. To the best of our knowledge, this is the first study to determine the efficacy of topical methylprednisolone cream in the early post-operative period for the prevention of scarring after cesarean section.

We have demonstrated a lower score on all MVSS parameters (height, pigmentation, vascularity, pliability, and total MVSS) at the 6^th^ month evaluation in both groups, with no significant differences between the groups. The most prominent changes were recorded in the height and vascularity score subsections of the MVSS at the 3^rd^ month assessment of the treatment group. Patient satisfaction was also higher in the experimental group which received methylprednisolone treatment.

Although intralesional corticosteroid injections have been preferred in some studies,^25^ they come with many adverse complications including pain, skin atrophy, changes in pigmentation, and the formation of white bead-like skin deposits. In our study, only two patients reported itching as an adverse effect after application of the methylprednisolone cream. Our study is the first clinical study involving corticosteroid application on Cesarean section scars. The inclusion of comparative and time-bound analyses are among the strengths of our study, as well as the 6-month individual follow-up time for each patient.

### Limitation of the study

We had relatively few participants which may have compromised statistical power. Furthermore, this is a study of wounds inflicted by a single operating surgeon; therefore, the specialty and experience of the surgeon is not evaluated. In addition, when allocating an experimental group with control group it should be considered that there are other potential methods to create the groups other than including age-matched counterparts. Repeating the methodology on a larger series of patients and trying different topical corticosteroid formulations could be considered in future studies.

## CONCLUSION

The clinical outcomes of individuals who did not receive any treatment and those who received corticosteroids were seen to be similar. Although positive effects were seen for certain outcomes at three months, similar final results were observed in the group that did not receive treatment at six months. This may have been due to the fact that treatment was ceased after three months, as the use of topical steroids is limited to 12 weeks in order to prevent side effects. We recommend that the hypothesis be tested in larger series in future studies.
